# Monitoring Volatility Change for Time Series Based on Support Vector Regression

**DOI:** 10.3390/e22111312

**Published:** 2020-11-17

**Authors:** Sangyeol Lee, Chang Kyeom Kim, Dongwuk Kim

**Affiliations:** Department of Statistics, Seoul National University, Seoul 08826, Korea; ckdk95@snu.ac.kr (C.K.K.); bitsaem@snu.ac.kr (D.K.)

**Keywords:** GARCH-type time series, CUSUM monitoring, support vector regression, particle swarm optimization

## Abstract

This paper considers monitoring an anomaly from sequentially observed time series with heteroscedastic conditional volatilities based on the cumulative sum (CUSUM) method combined with support vector regression (SVR). The proposed online monitoring process is designed to detect a significant change in volatility of financial time series. The tuning parameters are optimally chosen using particle swarm optimization (PSO). We conduct Monte Carlo simulation experiments to illustrate the validity of the proposed method. A real data analysis with the S&P 500 index, Korea Composite Stock Price Index (KOSPI), and the stock price of Microsoft Corporation is presented to demonstrate the versatility of our model.

## 1. Introduction

In this paper, we study the cumulative sum (CUSUM) monitoring procedure to sequentially detect a significant change in time series with conditional volatilities. Since [1,2], the CUSUM method has been an acclaimed tool to detect an anomaly among observations in statistical process control (SPC). In SPC, a control chart is a primary component that graphically describes the behavior of sequentially observed time series. For examples of SPC, see [3] for control charts, [4] for the analysis of complex environmental time series, and [5] for backcasting-forecasting time series. In particular, the CUSUM chart is one of the most frequently adopted methods among various fields of SPC. For an overview of SPC, we refer to [6]. In general, the performance of control charts is measured with average run length (ARL). However, instead of the conventional control charts, some authors, such as [7], alternatively took the approach of controlling type I errors in probability instead of controlling ARL to deal with the monitoring process in autoregressive time series. This design of the sequential monitoring method has merit in its ability to attain a lower false alarm rate, as seen in [8], who took a similar approach to dealing with generalized autoregressive conditional heteroscedastic (GARCH) time series. For more references as to the monitoring process in time series, see [9]. Here, inspired by the previous studies, we aim to hybridize the CUSUM monitoring scheme with support vector regression (SVR) for GARCH-type time series.

Support vector machine (SVM) is one of the most popular nonparametric learning methods used predominantly for classification and regression [10]. In particular, compared with traditional model-based methods, the support vector regression (SVR) is more advantageous to approximating the nonlinearity of the underlying dynamic structure of datasets. Refer to [11,12,13,14,15], and the papers cited therein. Moreover, SVR is structured to exploit the quadratic programming optimization problem, and to implement the structural risk minimization principle [16]. This facilitates SVR to be an estimation scheme that optimally minimizes the empirical risk while being relatively parsimonious [17]. This further enables the SVR to perform adequately for variously sized datasets, including the case where its size is relatively small. Refs. [18,19] recently used the SVR with a hybridization of the CUSUM method to detect a change point in SVR-autoregressive and moving average (ARMA) and SVR-GARCH models. Therein, the residual-based CUSUM test has been adopted by referring to the previous studies of [20,21,22]. See [23] for a general overview of the change point detection problem using CUSUM methods. In the same spirit, here we also take the approach of the CUSUM of squares test, rather than the score vector-based CUSUM test used in [7,8], as the former largely outperforms the latter in terms of stability and power, as seen in the empirical study of [21]; above all, the latter is only available for the model-based CUSUM test. As pointed out in the precedent works of [18,19], the role of the accurately computed residuals is substantially important, and thereby, so is the precise prediction of the used SVR method. As the optimal choice of tuning parameters matters to a significant degree as well, we consider adopting particle swarm optimization (PSO) [24] for the purpose of tuning parameter optimization. For a theoretical background, we refer to [25,26,27]. See also the survey papers of [28,29,30]. These are population-based algorithms and have been widely used to obtain optimal tuning parameters in SVR. Therefore, we evaluate the performance of the proposed SVR-GARCH-based CUSUM monitoring method equipped with the PSO through Monte Carlo experiments.

The rest of this paper is organized as follows. Section 2 introduces the CUSUM monitoring and explains its fundamental principle and application to GARCH-type time series. Section 3 describes SVR and PSO in general, then elaborates on the monitoring process for SVR-GARCH models in more detail. Section 4 presents Monte Carlo simulations conducted to evaluate the performance of the proposed method. Section 5 performs a real data analysis using the S&P 500 index, KOSPI, and the stock returns of Microsoft Corporation datasets. Finally, Section 6 provides concluding remarks.

## 2. CUSUM Monitoring Procedure

In this section, we introduce our monitoring process starting from the independent and identically distributed (iid) sample case. Let us consider the problem of monitoring an anomaly in the variance from a stream of observations $\epsilon_{t}$ of mean zero up to time *n*. Under the null hypothesis of no anomalies, we assume that $\epsilon_{t}$ are iid with unit variance over time t=1,…,n. Namely, we test

$$\begin{array}{ccc} & & H_{0}:\;\;\;Var\left(\epsilon_{t}\right)=1,\;\;t=1,\ldots ,n\;\;\;\mathrm{vs}.\\ & & H_{1}:\left\{\begin{array}{cc} Var(\epsilon_{t})=1, & t=1,\ldots ,k\\ Var(\epsilon_{t})\neq 1, & t=k+1,\ldots ,n \end{array}\right.\;\;\mathrm{for}\;\mathrm{some}\;k=2,\ldots ,n-1. \end{array}$$

For this task, we first define

(1) $$\begin{array}{c} W_{k}=\sum_{t=1}^{k}\left(\epsilon_{t}^{2}-1\right)/\tau , \end{array}$$

for each k≥1, where $\tau^{2}=Var\left(\epsilon_{1}^{2}\right)$, which is assumed to be known. Ref. [7] considered the monitoring process based on:

(2) $$\begin{array}{c} T_{n}^{\left(1\right)}=\max_{1\leq k\leq n}T_{n}\left(k\right)=\max_{1\leq k\leq n}\max_{m\leq k}\frac{1}{\sqrt{n}}\left(W_{m}-W_{k}\right), \end{array}$$

and later [8] additionally considered the monitoring process based on:

(3) $$\begin{array}{c} T_{n}^{{}^{\prime }}=\max_{1\leq k\leq n}T_{n}^{{}^{\prime }}\left(k\right)=\max_{1\leq k\leq n}\underset{1\leq m<m^{{}^{\prime }}\leq k}{\sup}\frac{1}{\sqrt{n}}\left|\frac{m}{m^{{}^{\prime }}}W_{m^{{}^{\prime }}}-W_{m}\right|. \end{array}$$

In this study, however, we consider another monitoring process based on

(4) $$\begin{array}{c} T_{n}^{max}=T_{n}^{\left(1\right)}\vee T_{n}^{\left(2\right)}:=\max\left\{T_{n}^{\left(1\right)},T_{n}^{\left(2\right)}\right\} \end{array}$$

with

(5) $$\begin{array}{c} T_{n}^{\left(2\right)}=\max_{1\leq k\leq n}T_{n}^{\left(2\right)}\left(k\right)=\max_{1\leq k\leq n}|\min_{m\leq k}\frac{1}{\sqrt{n}}\left(W_{m}-W_{k}\right)|. \end{array}$$

We employ the test statistic $T_{n}^{max}$ because it combines the strength of $T_{n}^{\left(1\right)}$ and $T_{n}^{\left(2\right)}$, which detect well the decrease and increase of variance, respectively. Note that if $E\epsilon_{k}^{2}$ has a positive shift, say, from 1 to 1+δ with δ>0, at some point $k_{0}=2,\ldots ,n-1$, $|\min_{m\leq k}W_{m}-W_{k}|$ for $k>k_{0}$ would tend to have larger values due to the shift, which, however, is not true for $\max_{m\leq k}W_{m}-W_{k}$, indicating that $T_{n}^{\left(2\right)}$ would be able to detect the shift well while $T_{n}^{\left(1\right)}$ would not do so; by contrast, if  $E\epsilon_{k}^{2}$ has a negative shift, $T_{n}^{\left(1\right)}$ would be able to detect the shift well while $T_{n}^{\left(2\right)}$ would not do so. Additionally, $T_{n}^{max}$ is preferable over $T_{n}^{{}^{\prime }}$ in (3) as the latter tends to detect well a change that occurs in the middle of time series.

Using Donsker’s invariance principle [31] and the fact that $\sup_{0\leq s\leq t}B\left(s\right)-B\left(t\right)=\left|B\left(t\right)\right|$ in distribution for any standard Brownian motion B, as described in [7], we have that as n→∞,

$$\begin{array}{ccc} & & T_{n}^{\left(1\right)}\overset{D}{\to }T=\underset{0\leq t\leq 1}{\sup}\left(\underset{0\leq s\leq t}{\sup}B\left(s\right)-B\left(t\right)\right)\overset{D}{=}\underset{0\leq t\leq 1}{\sup}\left|B\left(t\right)\right|,\\ & & T_{n}^{\left(2\right)}\overset{D}{\to }T^{{}^{\prime }}=\underset{0\leq t\leq 1}{\sup}\left(\underset{0\leq s\leq t}{\sup}\left(-B\right)\left(s\right)-\left(-B\right)\left(t\right)\right)\overset{D}{=}\underset{0\leq t\leq 1}{\sup}\left|B\left(t\right)\right|, \end{array}$$

where $\overset{D}{\to }$ denotes a convergence in distribution, and $X\overset{D}{=}Y$ implies the distribution functions of random variables *X* and *Y* being equivalent. Furthermore, by the continuous mapping theorem, $T_{n}^{max}$ in (4) satisfies

(6) $$\begin{array}{c} T_{n}^{max}\overset{D}{\to }T\vee T^{{}^{\prime }}. \end{array}$$

Then, the null hypothesis is rejected if $T_{n}^{max}$ is larger than a constant *c*, which is determined asymptotically as the number satisfying $P(T\vee T^{{}^{\prime }}>c)=\alpha$ for any significance α∈(0,1). In practice, we can obtain the critical value *c* empirically using Monte Carlo simulations. For example, c=2.46509 when α=0.05. Thus, an anomaly is signaled at *k* when $T_{n}^{max}\left(k\right)=T_{n}^{\left(1\right)}\left(k\right)\vee T_{n}^{\left(2\right)}\left(k\right)>c$ for some k=1,…,n.

This monitoring process can be extended to the GARCH(1,1) model [32]:

(7) $$\begin{array}{ccc} y_{t} & = & \sigma_{t}\epsilon_{t},\;\;t\in Z,\\ \sigma_{t}^{2} & = & \omega +\alpha y_{t-1}^{2}+\beta \sigma_{t-1}^{2} \end{array}$$

with ω>0,α≥0,β≥0 satisfying α+β<1 and $E\epsilon_{1}^{4}<\infty$. This model is widely used to model financial time series with high volatilities as it captures well the volatility clustering phenomenon. For its properties and characteristics and various GARCH variants, see [19,33], and the papers cited therein. Here, the monitoring process can be constructed based on residuals ${\hat{\epsilon }}_{t}=y_{t}/{\hat{\sigma }}_{t0}$ with ${\hat{\sigma }}_{t0}^{2}=\omega_{0}+\alpha_{0}y_{t-1,0}^{2}+\beta_{0}{\hat{\sigma }}_{t-1}^{2}$ with some initial values $y_{0}$ and $\sigma_{0}$ when the true parameters $\omega_{0},\alpha_{0},\beta_{0}$ are known in advance from past experience. However, when they are unknown, which is a prevalent phenomenon in practice, we instead construct the CUSUM test with residuals ${\hat{\epsilon }}_{t}=y_{t}/{\hat{\sigma }}_{t}$ with ${\hat{\sigma }}_{t}^{2}=\hat{\omega }+\hat{\alpha }y_{t-1}^{2}+\hat{\beta }{\hat{\sigma }}_{t-1}^{2}$, where $\hat{\omega },\hat{\alpha },\hat{\beta }$ are estimators of ω,α,β obtained from a given training sample. Then, we employ the following:

(8) $$\begin{array}{c} {\hat{T}}_{n}^{max}={\hat{T}}_{n}^{\left(1\right)}\vee {\hat{T}}_{n}^{\left(2\right)}, \end{array}$$

where ${\hat{T}}_{n}^{\left(i\right)}$ are the same as $T_{n}^{\left(i\right)}$ in (2) and (5), i=1,2, with $W_{k}$ in (1) replaced by ${\hat{W}}_{k}=\sum_{t=1}^{k}\left({\hat{\epsilon }}_{t}^{2}-{\bar{\hat{\epsilon }}}^{2}\right)/\hat{\tau }$, and ${\bar{\hat{\epsilon }}}^{2}$ and ${\hat{\tau }}^{2}$ are the sample mean and variance of the residuals obtained from the training sample, respectively. We reject the null hypothesis if ${\hat{T}}_{n}^{max}>c$ for the aforementioned critical value c>0. Theoretically, the limiting distribution of ${\hat{T}}_{n}^{max}$ is anticipated to be the same as the one in (6) when certain conditions are fulfilled, namely m=m(n) satisfies m/n→∞ as n→∞ (e.g., m∼cnloglogn for some c>0) and $\left(\hat{\omega },\hat{\alpha },\hat{\beta }\right)$ is a ($\sqrt{m}$-consistent) Gaussian quasi-maximum likelihood estimator (QMLE) as in [33], the proof of which is rather standard and omitted for brevity. The monitoring process of the nonlinear time series via SVR is provided in Section 3.3 below.

## 3. Monitoring Procedure via SVR-GARCH Model

### 3.1. Support Vector Regression

Support vector regression (SVR) is a nonparametric function estimating method, which is a branch of the support vector machine that originated from [34] as a classification tool. Here, SVR is utilized to conduct CUSUM tests, as it effectively incorporates circumstances where the underlying structure of the conditional variance presented in (7) is unknown, and possibly nonlinear. Its details will be elaborated in Section 3.3 below.

In SVR, we seek to find a function of the form:

f(x)=〈w,ϕ(x)〉+b,

where *x* denotes a vector of explanatory variables, *w* and *b* are regression parameters to be estimated, and ϕ is an implicit kernel operator that satisfies $K\left(x_{1},x_{2}\right)=\phi \left(x_{1}\right)\phi \left(x_{2}\right)$ for some pre-defined kernel function *K*.

To obtain the estimates of the regression parameters, we then formulate the problem where we can exploit its structure to employ a quadratic programming method [17]:

(9) $$\begin{array}{c} \mathrm{minimize}\;\frac{1}{2}{\left||w|\right|}^{2}+C\sum_{i=1}^{n}\left(\xi_{i}+\xi_{i}^{*}\right), \end{array}$$

$$\mathrm{subject}\;\mathrm{to}\;\left\{\begin{array}{c} y_{i}-\left\{w^{T}\phi \left(x_{i}\right)+b\right\}\leq \epsilon +\xi_{i}^{*}\\ \left\{w^{T}\phi \left(x_{i}\right)+b\right\}-y_{i}\leq \epsilon +\xi_{i}\\ \xi_{i}\geq 0,\;\xi_{i}^{*}\geq 0, \end{array}\right.$$

where $\xi_{i},\xi_{i}^{*}>0$ denote slack variables, C>0 denotes a penalty term, and ϵ is a tuning parameter that determines the level of tolerance regarding the error. Note that *C* and ϵ are user-defined tuning parameters of the model.

By the duality of the Karush–Khun–Tucker condition, we obtain the following optimization problem with respect to the Lagrange multipliers $\alpha_{i}$ and $\alpha_{i}^{*}$ as dual variables [16]:

(10) $$\begin{array}{cc} \mathrm{maximize}\; & -\frac{1}{2}\sum_{i=1}^{n}\sum_{j=1}^{n}\left(\alpha_{i}-\alpha_{i}^{*}\right)\left(\alpha_{j}-\alpha_{j}^{*}\right)\phi {\left(x_{i}\right)}^{T}\phi \left(x_{j}\right)\\ \; & -\epsilon \sum_{i=1}^{n}\left(\alpha_{i}+\alpha_{i}^{*}\right)+\sum_{i=1}^{n}\left(\alpha_{i}-\alpha_{i}^{*}\right)y_{i}, \end{array}$$

$$\mathrm{subject}\;\mathrm{to}\;\left\{\begin{array}{c} \sum_{i=1}^{n}\left(\alpha_{i}-\alpha_{i}^{*}\right)=0\\ 0\leq \alpha_{i}\leq C,\;0\leq \alpha_{i}^{*}\leq C. \end{array}\right.$$

Consequently, the solutions of the optimization problem (10) constructs the estimate $\hat{f}$ of *f* as follows:

$$\begin{array}{cc} \hat{w} & =\sum_{i=1}^{n}\left(\alpha_{i}-\alpha_{i}^{*}\right)\phi \left(x_{i}\right),\\ \hat{f}\left(x\right) & =\sum_{i=1}^{n}\left(\alpha_{i}-\alpha_{i}^{*}\right)K\left(x_{i},x\right)+\hat{b}, \end{array}$$

where $\hat{b}$ can be obtained with various approaches. See [17] or [35] for references.

The tuning parameters of our SVR-GARCH model consist of C,ϵ, and $\gamma^{2}$, as we utilize the Gaussian kernel function

$$K\left(x,y\right)=\exp\left(-\frac{||x-{y||}^{2}}{2\gamma^{2}}\right).$$

Moreover, as the tuning parameters are user-defined, it must either be selected prior to employing the SVR, or be optimized. Here, we utilize the PSO to select the set of tuning parameters. The procedure of the PSO algorithm is elucidated in more detail in the next section.

### 3.2. Particle Swarm Optimization

PSO is one of the widely acclaimed meta-heuristic methodologies and is extensively adopted when optimizing tuning parameters in various machine learning techniques. Initially proposed by [24], its ability of optimization in nonlinear and nonconvex problems is inspired by the movement of organisms in bird flocks or animal herds. Here, we refer to [30] to describe the procedure.

Given a suitable objective function, a standard PSO algorithm aims to optimize a *d*-dimensional parameter *x* in the following search space:

$$X=\{x={\left(x_{1},\ldots ,x_{d}\right)}^{T}\in R^{d}:l_{k}\leq x_{k}\leq u_{k},\;k=1,\cdots d\},\;d\geq 1,$$

for some $u_{k},l_{k}\in R$, k=1,⋯d. A particle *j* at time *t*, denoted by a *d*-dimensional vector $x_{j}\left(t\right)$ in X with

$$x_{j}\left(t\right)={\left(x_{j1}\left(t\right),x_{j2}\left(t\right),\cdots ,x_{jd}\left(t\right)\right)}^{T}\in X,$$

is considered as a candidate of the set of optimal solutions. Moreover, a swarm at time *t* is defined as $S\left(t\right)={\left(x_{1}\left(t\right),\cdots ,x_{N}\left(t\right)\right)}^{T}$. Each particle *j* has its own *d*-dimensional velocity vector at time *t*, denoted by $v_{j}\left(t\right)\in V$ for j=1,⋯N, where

$$V=\{v={\left(v_{1},\ldots ,v_{d}\right)}^{T}\in R^{d}:v_{min}\leq v_{k}\leq v_{max},k=1,\cdots ,d\}$$

is a velocity space with $v_{min}$ and $v_{max}$ being the lower and upper bound of each velocity element, respectively [36]. Furthermore, the best optimal solution $p_{j}\left(t\right)$ is defined as:

$$p_{j}\left(t\right)={\left(p_{i1},\cdots ,p_{jd}\right)}^{T}\in X_{\left(j,t\right)},$$

where $X_{\left(j,t\right)}:=\left\{x_{j}\left(1\right),\cdots ,x_{j}\left(t\right)\right\}$ denotes the trajectory of the particle *j* until time *t*.

Then, the global best optimal solution of the trajectory of all particles until time *t* is defined as $g\left(t\right)={\left(g_{1},\cdots ,g_{d}\right)}^{T}\in \left\{p_{j}\left(1\right),\ldots ,p_{j}\left(t\right)\right\}$. In each generation at time $t\in \left[1,T_{max}\right]$, where $T_{max}$ is a prescribed maximum generation time, the *j*-th particle in swarm S(t) is updated as follows:

$$\begin{array}{cc} v_{j}\left(t+1\right) & =w\left(t\right)v_{k}\left(t\right)+c_{1}z_{1}\left(p_{i}\left(t\right)-x_{i}\left(t\right)\right)+c_{2}z_{2}\left(g\left(t\right)-x_{i}\left(t\right)\right)\\ x_{i}\left(t+1\right) & =x_{i}\left(t\right)+v_{i}\left(t+1\right), \end{array}$$

where $c_{1},c_{2}$ are acceleration factors in R and $z_{1},z_{2}$ are random variables generated from a uniform distribution U[0,1]. In addition, w(t) is an inertia term at time *t*, which is calculated as follows:

$$w\left(t\right)=\left(w_{\mathrm{start}}-w_{\mathrm{end}}\right)\left(\frac{T_{\max}-t}{T_{\max}}\right)+w_{\mathrm{end}},$$

where $w_{\mathrm{start}}$ and $w_{\mathrm{end}}$ are predefined lower and upper bounds of the inertia values, respectively. Ref. [30] recently proved that the optimal solution obtained from the PSO algorithm converges to the global optimum with probability 1 under regularity conditions.

The process of the aforementioned PSO algorithm is condensed in Algorithm 1 below.
**Algorithm 1** Standard PSO algorithm1: **procedure**
PSO($N,w_{\max},w_{\min},c_{1},c_{2},T_{\max}$)2:
$\mathrm{Accelerated}\;\mathrm{factor}=\left(c_{1},c_{2}\right),\max\;\mathrm{generation}\;=T_{\max}$3:  Initializelocationandvelocity;4:  **while**
$t<T_{\max}$
**do**5:    t←t+1;6:    $w\left(t\right)\leftarrow w_{\max}-\frac{w_{\max}-w_{\min}}{T_{\max}}$7:    **for**
i=1,…,N
**do**8:     update $v_{i}\left(t\right)$;9:     $x_{i}\left(t\right)\leftarrow x_{i}\left(t-1\right)+v_{i}\left(t\right)$;10:     update $p_{i}\left(t\right);$11:    **end for**12:    update g(t);13:  **end while**14: **end procedure**


### 3.3. Monitoring Nonlinear Time Series via SVR

We extend the monitoring process introduced in Section 2 to embrace nonlinear GARCH models with the following form:

(11) $$\begin{array}{c} \left\{\begin{array}{cc} y_{t} & =\sigma_{t}\epsilon_{t},\\ \sigma_{t}^{2} & =g(y_{t-1}^{2},\sigma_{t-1}^{2}), \end{array}\right. \end{array}$$

where *g* is an unknown, possibly nonlinear, function to be estimated, $\sigma_{t}^{2}$ is the conditional volatility, and $\epsilon_{t}$ are iid errors with zero mean and unit variance. To obtain the residuals that formulate the test statistic (8) of the monitoring process, we estimate *g* by $\hat{g}$ with a training sample $y_{1},\ldots ,y_{m}$. For the estimation procedure, we utilize the SVR and then adopt the notion of the retrospective test described in [19].

For estimating $\hat{g}$ via SVR, we first divide the training sample into two distinct samples, say $\left\{y_{1},\ldots ,y_{l}\right\}$ and $\left\{y_{l+1},\ldots ,y_{m}\right\}$, then regard the latter as the validation set. Subsequently, we replace $\sigma_{t}^{2}$ with its proxy ${\tilde{\sigma }}_{t}^{2}$, as $\sigma_{t}^{2}$ is unknown in practice. The authors of [14] employed the moving average method with a window size s≥1 as below:

$${\tilde{\sigma }}_{t}^{2}=\sum_{j=1}^{s}y_{t-j+1}^{2}$$

for t=1,…,n, where $y_{0},y_{-1},\ldots ,y_{-m+1}$ are some sensible initial values. Although its validity is advocated when s=5 in [19], we alternatively consider the exponentially-weighted moving average (EWMA) estimator

$$\begin{array}{c} {\tilde{\sigma }}_{t}^{2}=\alpha {\tilde{\sigma }}_{t-1}^{2}+\left(1-\alpha \right)y_{t}^{2}, \end{array}$$

where α∈(0,1) is a tuning parameter, which we set to 0.94 in our study, and ${\tilde{\sigma }}_{0}^{2}>0$ is some initial value. This alteration improves both the stability and the performance of the model, as portrayed in Section 4.

Moreover, to further enhance the stability of the estimation process of $\hat{g}$, we log-transform the response variable of (11), then take the exponential to obtain ${\hat{\sigma }}_{t}^{2}$. To elaborate, we recursively obtain ${\hat{\sigma }}_{t}^{2}$ through $\gamma_{t}:=\log\sigma_{t}^{2}$ as follows:

(12) $$\begin{array}{cc} \gamma_{t} & =g^{*}\left(y_{t-1}^{2},\sigma_{t-1}^{2}\right),\;g^{*}=\log g,\\ {\hat{\gamma }}_{t} & ={\hat{g}}^{*}\left(y_{t-1}^{2},{\tilde{\sigma }}_{t-1}^{2}\right)\leftarrow {\tilde{\gamma }}_{t}=\log{\tilde{\sigma }}_{t}^{2},\\ {\hat{\sigma }}_{t}^{2} & =\exp\left({\hat{\gamma }}_{t}\right). \end{array}$$

Given $\hat{g}$ and a space of tuning parameters Θ, we then employ the PSO algorithm to obtain an optimal set of tuning parameters $\theta^{*}\in \Theta$ by evaluating the mean absolute error (MAE):

$$\mathrm{MAE}=\frac{1}{m-l}\sum_{t=l+1}^{m}\left|{\hat{\sigma }}_{t}^{2}-{\tilde{\sigma }}_{t}^{2}\right|,$$

where ${\hat{\sigma }}_{t}^{2}$ and ${\tilde{\sigma }}_{t}^{2}$ are obtained with a validation time series. Ultimately, the finalized $\hat{g}$ is obtained by utilizing all the training samples $\left\{y_{1},\ldots ,y_{m}\right\}$ and $\theta^{*}$.

The test set of length *n* emerges sequentially in practice, denoted by $y_{m+1},\ldots ,y_{m+n}$. Then, utilizing $\hat{g}$, we yield the residuals

$${\hat{\epsilon }}_{t}=y_{t}/{\hat{\sigma }}_{t},$$

where ${\hat{\sigma }}_{t}^{2}$ is obtained recursively through (12). Afterwards, upon observing $y_{l},\;m+1\leq l\leq m+n$, the monitoring procedure is conducted by computing the CUSUM test statistic as in (8), and we declare it out of control when it exceeds the prescribed critical value under the nominal level α∈(0,1).

**Remark** **1.** *In our proposed monitoring procedure, we use the theoretically obtained critical value c, as illustrated by its notable performance in Section 4. However, if m is not large enough relative to n, there is a chance that the monitoring procedure might be undermined by the parameter estimation. In this case, one may be able to obtain c empirically through a wild bootstrap approach with the following steps [21]:*

*1.*  *Estimate ω,α,β with $\hat{\omega },\hat{\alpha },\hat{\beta }$ from training sample $y_{1},\ldots ,y_{m}$;*
*2.*  *Estimate $\sigma_{t}^{2}$ recursively with ${\hat{\sigma }}_{t}^{2}=\hat{\omega }+\hat{\alpha }y_{t-1}^{2}+\hat{\beta }{\hat{\sigma }}_{t-1}^{2}$ and some initial values $y_{0}$ and ${\hat{\sigma }}_{0}$;*
*3.*  *Generate iid standard normal random variables $\eta_{t}^{b}$, t=1,…,n, b=1,…,B, and construct a bootstrap sample $y_{t}^{b}={\hat{\sigma }}_{t}\eta_{t}^{b}$;*
*4.*  Based on $y_{t}^{b}$, t=1,…,n, estimate ω,α,β with ${\hat{\omega }}^{*},{\hat{\alpha }}^{*},{\hat{\beta }}^{*}$, and calculate the bootstrapped residuals ${\hat{\epsilon }}_{t}^{b*}=y_{t}^{b}/{\hat{\sigma }}_{t}^{b*}$ with ${\hat{\sigma }}_{t}^{b*}$ obtained recursively by ${\hat{\sigma }}_{t}^{b*2}={\hat{\omega }}^{*}+{\hat{\alpha }}^{*}y_{t-1}^{b2}+{\hat{\beta }}^{*}{\hat{\sigma }}_{t-1}^{b*2}$;
*5.*  *Based on these residuals, construct the monitoring process ${\hat{T}}_{n}^{max,b}\left(k\right)$, k=1,…,n, b=1,…,B, similarly to ${\hat{T}}_{n}^{max}$ in (8) with ${\hat{W}}_{k}^{b}$ analogously defined to ${\hat{W}}_{k}$;*
*6.*  *Finally, the critical value c is determined as the 100α% upper quantile of ${\hat{T}}_{n}^{max,b}=\max_{1\leq k\leq n}{\hat{T}}_{n}^{max,b}\left(k\right)$ for b=1,…,B.*

The critical value *c* can be obtained via a bootstrap method similar to that for the GARCH(1,1) model in (7). In this case, we use $y_{t}^{b}={\tilde{\sigma }}_{t}\eta_{t}$. Based on the obtained bootstrap sample $y_{t}^{b}$, applying the SVR method again, we can get the conditional volatility estimates ${\hat{\sigma }}_{t}^{*b2}={\hat{g}}^{*}\left(y_{t-1}^{b2},{\tilde{\sigma }}_{t-1}^{b2}\right)$, where ${\tilde{\sigma }}_{t}^{b2}$ are proxies obtained from $y_{t}^{b}$’s. Then, the bootstrapped residuals are obtained as ${\hat{\epsilon }}_{t}^{b*}=y_{t}^{b}/{\hat{\sigma }}_{t}^{*b}$. The critical value is obtained from these similarly to Steps (5) and (6) addressed above. Instead of ${\hat{\sigma }}_{t}^{*b2}$, alternatively one might be able to consider using ${\tilde{\sigma }}_{t}^{*b2}:=\hat{g}\left(y_{t-1}^{b2},{\tilde{\sigma }}_{t-1}^{b2}\right)$ to obtain the residuals ${\hat{\epsilon }}_{t}^{b*}:=y_{t}^{b}/{\tilde{\sigma }}_{t}^{*b}$.

## 4. Simulation Experiments

We assess the proposed SVR-GARCH monitoring process to measure the performance when the time series is simulated from linear or nonlinear variants of GARCH models, such as GARCH(p,q), asymmetric GARCH(p,q) (AGARCH), GJR-GARCH(p,q), and Box–Cox transformed threshold GARCH(p,q) (BCTT-GARCH), specified as follows:

$$\begin{array}{cc} \mathrm{GARCH}\left(p,q\right):\;\;\;y_{t} & =\sigma_{t}\epsilon_{t},\\ \sigma_{t}^{2} & =\omega +\sum_{i=1}^{p}\alpha_{i}y_{t-i}^{2}+\sum_{j=1}^{q}\beta_{j}\sigma_{t-j}^{2},\\ \alpha_{i} & \geq 0,\beta_{j}\geq 0;\\ \mathrm{AGARCH}\left(p,q\right):\;\;\;y_{t} & =\sigma_{t}\epsilon_{t},\\ \sigma_{t}^{2} & =\omega +\alpha {\left(y_{t-1}-b\right)}^{2}+\beta \sigma_{t-1}^{2},\\ \alpha & \geq 0,\beta \geq 0;\\ \mathrm{GJR}-\mathrm{GARCH}\left(p,q\right):\;\;\;y_{t} & =\sigma_{t}\epsilon_{t},\\ \sigma_{t}^{2} & =\omega +\sum_{i=1}^{p}\left(\alpha_{1,i}{y_{t-i}^{+}}^{2}+\alpha_{2,i}{y_{t-i}^{-}}^{2}\right)+\sum_{j=1}^{q}\beta_{j}\sigma_{t-j}^{2},\\ y_{t}^{+} & =\max\left(y_{t},\;0\right),\;y_{t}^{-}=-\min\left(y_{t},\;0\right),\\ \alpha_{1,i} & \geq 0,\alpha_{2,i}\geq 0,\beta_{j}\geq 0; \end{array}$$

$$\begin{array}{cc} \mathrm{BCTT}-\mathrm{GARCH}\left(p,q\right):\;\;\;y_{t} & =\sigma_{t}\epsilon_{t},\\ \sigma_{t}^{2} & ={\left[\omega +\sum_{i=1}^{p}\left\{\alpha_{1,i}{\left({y_{t-i}^{+}}^{2}\right)}^{\delta }+\alpha_{2,i}{\left({y_{t-i}^{-}}^{2}\right)}^{\delta }\right\}+\sum_{j=1}^{q}\beta_{j}\sigma_{t-j}^{2}\right]}^{1/\delta },\\ y_{t}^{+} & =\max\left(y_{t},\;0\right),\;y_{t}^{-}=-\min\left(y_{t},\;0\right),\\ \alpha_{1,i} & \geq 0,\alpha_{2,i}\geq 0,\beta_{j}\geq 0, \end{array}$$

where the orders p,q are fixed as 1 and the errors $\epsilon_{t}$ are iid. N(0,1) are random variables.

In implementation, we generate a time series of length *m* from each of the above models and fit the SVR-GARCH model to it as presented in Section 3. In this procedure, we utilize the first $\left\lfloor 0.7m\right\rfloor$ time series as a training set, and the rest as a validation set, where $\left\lfloor x\right\rfloor$ denotes the largest integer not exceeding *x*. Subsequently, to create the circumstance, where the dataset for monitoring (testing sample) is observed sequentially, we initially design the underlying parametric model with a change located at point 1<s<n. With this model, we generate a single observation of time series, obtain the residuals with the trained SVR-GARCH model, and then compute the test statistic. We terminate this monitoring process either if the test statistics are larger than the critical value *c*, or the accumulated number of observations reaches the prescribed length of *n*. To obtain the sizes and powers empirically, we iterate the procedure 1000 times at the significance level of α=0.05. The empirical sizes and powers are calculated as the ratio of the rejections of the null hypothesis $H_{0}$ out of the 1000 repetitions.

Upon the construction of the null hypothesis, we consider the following settings:

1. GARCH(1,1): ω=0.3,α=0.3,β=0.3
2. AGARCH(1,1): ω=0.1,α=0.1,β=0.8,b=1
3. GJR-GARCH(1,1): $\omega =0.1,\;\alpha_{1}=0.3,\;\alpha_{2}=0.1,\;\beta =0.5$
4. BCTT-GARCH(1,1): $\omega =0.3,\;\alpha_{1}=0.4,\;\alpha_{2}=0.2,\;\beta =0.3,\;\delta =0.8$.

We inspect the cases of (m,n)=(1000,1000) and (2000,2000), where $m=\left\lfloor cn\log\log n\right\rfloor$ is used, i.e., c=1.933,2.028 for n=1000,2000, respectively, and count the number of false alarms and anomaly detections when the underlying model experiences a change at diverse locations, namely at the observation $\left\lfloor \gamma n\right\rfloor$ of the monitoring time series for γ∈{0.1,0.2,…,0.7}. For each set of experiments, we employ the PSO search algorithm for optimizing tuning parameters.

**Figure 1:**
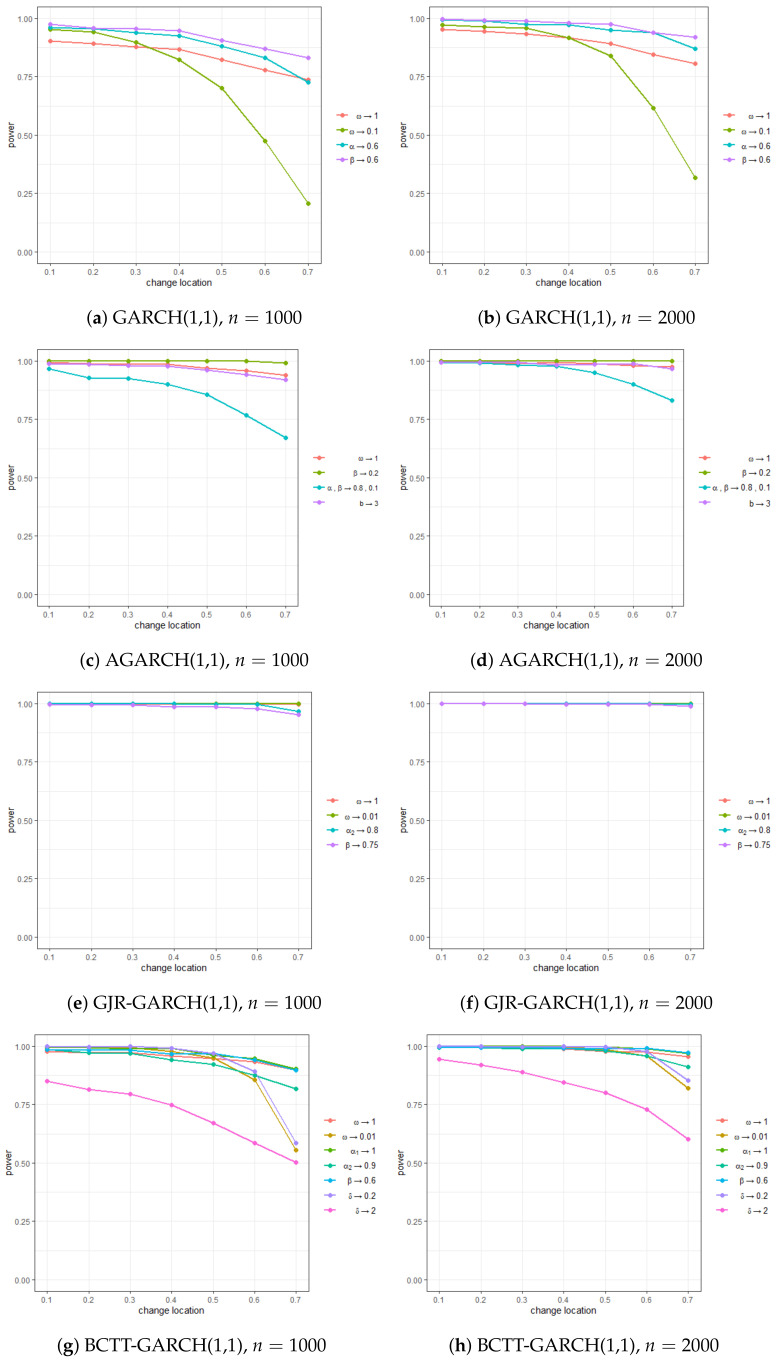
Plots of empirical power of the SVR-GARCH monitoring procedure.

Table 1 and Figure 1 encapsulate the empirical sizes and powers for the above four cases. In a nutshell, all four models’ empirical sizes show a tendency to stabilize around α=0.05. Additionally, their empirical powers approached 1 in a vast majority of the experiments, exhibiting a remarkable ability to detect changes across the experiments. In particular, as anticipated, the power became more significant as the γ decreases. Nevertheless, even if the location of the change was towards the end of the observation (i.e., a larger γ), its detection ability was still not heavily deteriorated. This entails that the performance of our monitoring process is not much affected by the location of change.

The result for the AGARCH model, presented in Figure 1c,d, is particularly more prominent. Despite the model being systematically unstable because of α+β being close to 1, empirical sizes and powers appeared to be highly reliable even at a relatively small sample size of n=1000. This robustly confirms the validity of our proposed method. Overall, our findings show that the proposed monitoring process is highly applicable to datasets with high volatilities, such as the daily returns of financial time series.

## 5. Real Data Analysis

In this section, we demonstrate the real-world applicability of our proposed SVR-GARCH monitoring scheme with three financial time series: the S&P500 Composite Index (2 January 1991∼31 December 2003), the Korea Composite Stock Price Index (KOSPI) (2 July 2012∼29 September 2020), and the stock price of Microsoft Corporation (1 July 2009∼30 September 2020), obtainable from the websites Yahoo Finance and Investing.com. Moreover, the log returns, defined as $r_{t}=100\times \left\{\log\left(y_{t}\right)-\log\left(y_{t-1}\right)\right\}\;\left(t\geq 2\right)$, are utilized throughout the analysis, and are denoted by “S&P500”, “KOSPI”, and “Microsoft”.

**Figure 2:**
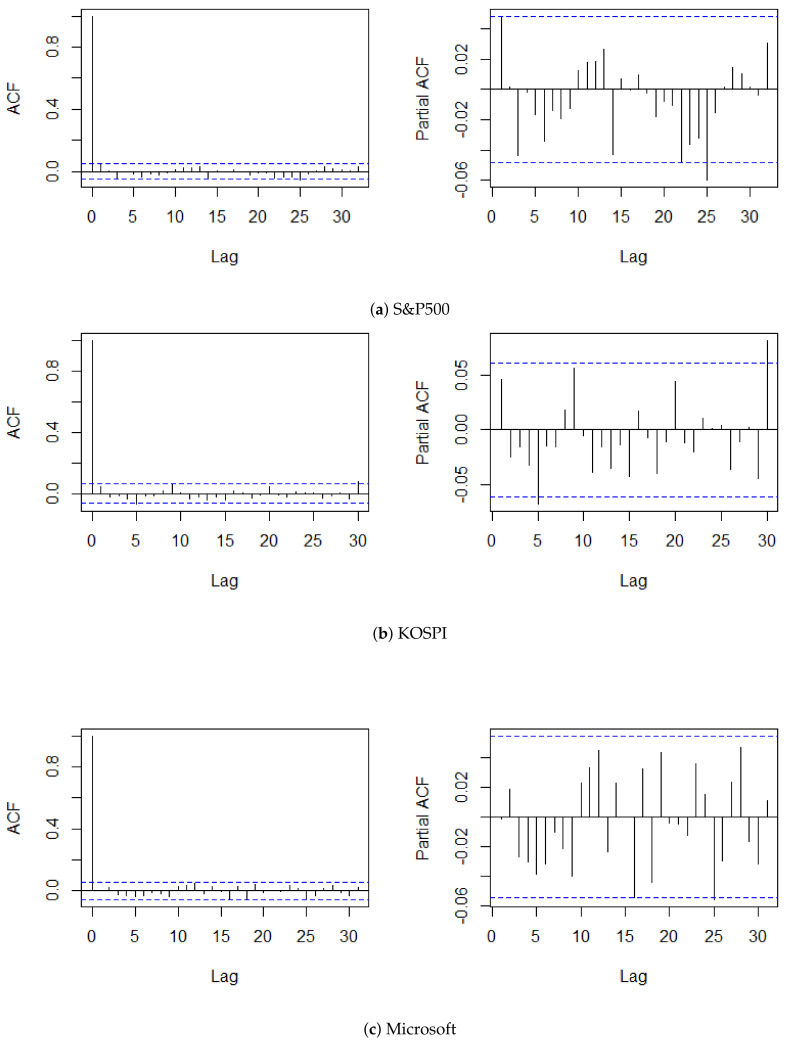
Plot of ACF and PACF up to lag 25 of log-returns.

The procedure of this analysis is categorized into four steps. We first divided the time series of log returns into two chunks, and assigned the former as the training set of size *m*. To reflect the situation of monitoring, setting the maximum number of observations to n=1500, we regarded the latter time series being observed sequentially. We then overviewed the general behavior of the given time series with its summary statistics, autocorrelation functions (ACFs), and partial ACFs (PACFs). Table 2 and Figure 2 reveal the basic characteristics of the datasets and plots of the ACFs and PACFs up to lag 25, respectively. The characteristics of KOSPI is similar to those of the standard normal distribution, in terms of skewness and excess kurtosis. By contrast, the excess kurtosis of S&P500 and Microsoft drifted from that of a standard normal distribution, and was moderately skewed, compared to KOSPI. Moreover, the ACFs and PACFs of all three time series did not suggest significant autocorrelations, which entails the stationarity of the datasets. Additionally, fitting the SVR-GARCH model of Lee et al. (2020b) to examine an existence of change within the training time series, we applyed their retrospective CUSUM test to the training set. Then, the result exhibited that it did not exceed the critical value of 1.3397, which consolidated the absence of change within the training set at the significance level of 0.05.

**Figure 3:**
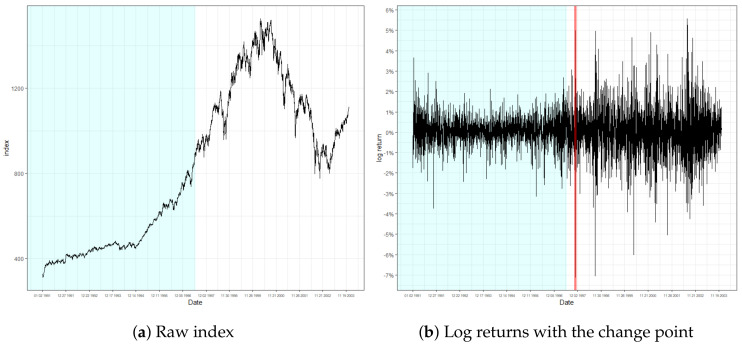
Raw index and its log returns of S&P500.

**Figure 4:**
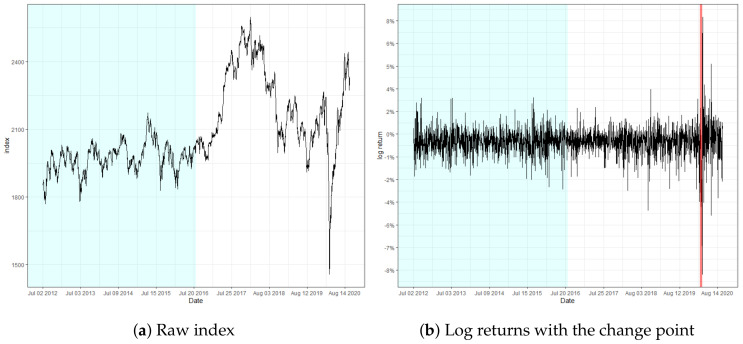
Raw index and its log returns of KOSPI.

**Figure 5:**
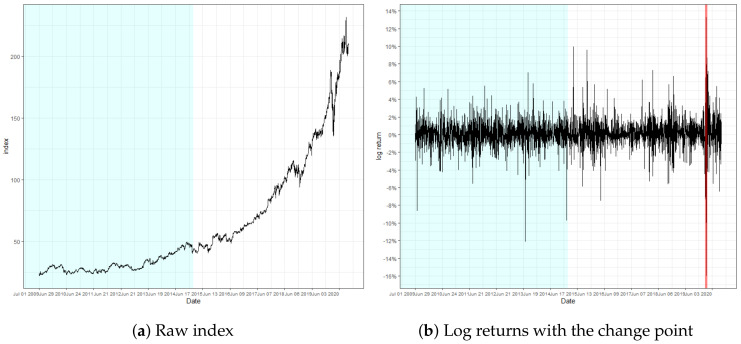
Raw index and its log returns of Microsoft.

Figure 3, Figure 4 and Figure 5 present the result of the monitoring procedure and the identified location of change in conditional volatility. The change of volatility for S&P500 appeared to occur on 28 October 1997. Notably, our model promptly responded to the event that occurred on the day prior, where log returns fluctuated by more than 7%. Additionally, we can observe the increase of volatility posterior to the detected location. Indeed, the detected location is around the period of the Asian financial crisis of 1997, and specifically on the exact date when the South Korean won plummeted massively to its new low. This instantaneous response after an abrupt change of volatility strengthens the validity of our monitoring process.

The locations of change regarding KOSPI and Microsoft were observed on 11 March and 13 March 2020, respectively. Notice that the detected location of change is close to the predetermined *n*. These findings particularly implicate that the detection ability of our monitoring process is not impaired, even if the volatility change is located near the end. In addition, the change point in KOSPI predates the major spread of the global pandemic outbreak by a few days, where the log-returns are observed to fluctuate abruptly, exceeding 8%. In fact, the recognized locations of change regarding KOSPI and Microsoft are around the period of the stock market crash resulting from the pandemic, which still currently affects the global financial market to a certain extent. This result illuminates the potential of our monitoring scheme to identify a signal change prior to the change in the underlying structure of the model and to serve as a solution to the necessity of prompt detection of structural changes throughout various fields of research.

## 6. Concluding Remarks

In this study, we considered a novel monitoring process for detecting a significant change of conditional volatilities in time series. For this task, we proposed a procedure based on a CUSUM test with a new test statistic, and utilized the SVR-GARCH model to calculate the residuals so as to construct the monitoring process, wherein the PSO is employed to obtain an optimal set of tuning parameters. Our simulation study demonstrated the adequacy of the proposed monitoring process for various linear and nonlinear GARCH-type time series. A real data analysis of three financial time series, namely S&P500, KOSPI, and Microsoft, was conducted to affirm the practicability of our model in various real-world circumstances. Our proposed model can be adopted in a classical SPC, where one controls ARL directly rather than the Type I error. This could be empirically achieved using the bootstrap method, as stated in Section 3.3. As the necessity for control charts with their usage tailored to various circumstances is still increasing, we leave the issue as our future research project.

## Figures and Tables

**Table 1:** Empirical size and power of the SVR-GARCH monitoring procedure for the GARCH(1,1), AGARCH(1,1), GJR-GARCH(1,1), and BCTT-GARCH(1,1) models.

4-17			n=1000							n=2000						
Change location			0.1n	0.2n	0.3n	0.4n	0.5n	0.6n	0.7n	0.1n	0.2n	0.3n	0.4n	0.5n	0.6n	0.7n
GARCH(1,1)	size		0.038							0.045						
	power	ω→1	0.903	0.893	0.879	0.867	0.824	0.778	0.737	0.953	0.945	0.934	0.916	0.893	0.845	0.805
		ω→0.1	0.954	0.942	0.898	0.824	0.701	0.475	0.206	0.971	0.963	0.959	0.916	0.840	0.616	0.317
		α→0.6	0.961	0.955	0.940	0.924	0.882	0.830	0.726	0.995	0.990	0.976	0.973	0.951	0.939	0.870
		β→0.6	0.974	0.958	0.955	0.946	0.907	0.871	0.832	0.996	0.992	0.988	0.981	0.975	0.940	0.920
AGARCH(1,1)	size		0.039							0.037						
	power	ω→1	0.993	0.989	0.987	0.985	0.968	0.959	0.939	0.996	0.997	0.988	0.995	0.989	0.980	0.975
		β→0.2	1.000	1.000	1.000	1.000	1.000	1.000	0.992	1.000	1.000	1.000	1.000	1.000	1.000	1.000
		α,β→0.8,0.1	0.967	0.927	0.924	0.900	0.856	0.767	0.672	0.995	0.992	0.983	0.978	0.950	0.899	0.832
		b→3	0.989	0.986	0.981	0.978	0.962	0.943	0.920	0.994	0.995	0.994	0.984	0.987	0.988	0.966
GJR-GARCH(1,1)	size		0.042							0.033						
	power	ω→1	1.000	0.999	1.000	0.998	0.999	0.998	0.996	1.000	1.000	1.000	1.000	0.999	1.000	0.999
		ω→0.01	1.000	1.000	1.000	1.000	1.000	1.000	1.000	1.000	1.000	1.000	1.000	1.000	1.000	1.000
		$\alpha_{2}\to 0.8$	1.000	1.000	0.999	0.999	0.998	0.997	0.967	1.000	1.000	1.000	1.000	0.999	1.000	0.998
		β→0.75	0.996	0.993	0.994	0.986	0.986	0.977	0.954	1.000	1.000	1.000	0.998	0.998	0.997	0.989
BCTT-GARCH(1,1)	size		0.048							0.039						
	power	ω→1	0.978	0.973	0.973	0.957	0.948	0.934	0.897	0.997	0.996	0.993	0.989	0.979	0.975	0.955
		ω→0.01	0.995	0.995	0.993	0.978	0.951	0.855	0.556	0.999	0.997	0.997	0.997	0.987	0.958	0.819
		$\alpha_{1}\to 1$	0.997	0.995	0.988	0.992	0.962	0.947	0.903	1.000	0.999	1.000	1.000	0.996	0.989	0.968
		$\alpha_{2}\to 0.9$	0.987	0.971	0.968	0.941	0.921	0.875	0.817	0.995	0.993	0.988	0.992	0.980	0.958	0.911
		β→0.6	0.986	0.983	0.984	0.967	0.970	0.941	0.897	0.998	0.994	0.997	0.992	0.990	0.992	0.972
		δ→0.2	0.999	0.998	0.999	0.992	0.969	0.891	0.585	0.999	0.999	0.998	0.999	0.996	0.978	0.854
		δ→2	0.850	0.814	0.795	0.749	0.671	0.585	0.502	0.945	0.919	0.890	0.846	0.800	0.728	0.602

**Table 2:** Summary statistics and the results of the preliminary retrospective test of the training set, and the result of the monitoring test regarding S&P500, KOSPI, and Microsoft.

		S&P500	KOSPI	Microsoft
Summary statistics (training set)	Observations	1640	1016	1417
	Mean	0.0604	0.0096	0.0428
	Standard deviation	0.6931	0.7728	1.4408
	Minimum	−3.7272	−3.1429	−12.1033
	Median	0.0352	0.0070	0.03145
	Maximum	3.6642	2.9124	7.0330
	Skewness	−0.1064	−0.0264	−0.6141
	Excess kurtosis	2.2428	1.3848	6.8293
Retrospective test (training set)	Test statistic	0.8069	1.2876	0.5897
Monitoring test	Location	97/10/28	20/03/11	20/03/13

## Abbreviations

The following abbreviations are used in this manuscript:

CUSUM	cumulative sum
SPC	statistical process control
ARL	average run length
ARMA	autoregressive and moving average
ARCH	autoregressive conditionally heteroskedasticity
GARCH	generalized autoregressive conditionally heteroskedasticity
SVR	support vector regression
SVM	support vector machine
PSO	particle swarm optimization
iid	independent and identically distributed
MAE	mean absolute error
EWMA	exponentially weighted moving average
AGARCH	asymmetric GARCH
GJR-GARCH	Glosten, Jagannathan and Runkle-GARCH
BCTT-GARCH	Box-Cox transformed threshold GARCH
ARMA	autoregressive and moving average
QMLE	quasi-maximum likelihood estimator
KOSPI	Korea Composite Stock Price Index
ACF	autocorrelation function
PACF	partial ACF
